# Derivation of neural stem cells from an animal model of psychiatric disease

**DOI:** 10.1038/tp.2013.96

**Published:** 2013-11-05

**Authors:** A de Koning, N M Walton, R Shin, Q Chen, S Miyake, K Tajinda, A K Gross, J H Kogan, C L Heusner, K Tamura, M Matsumoto

**Affiliations:** 1 Free University Medical Center, Amsterdam, HZ, The Netherlands; 2 CNS, Astellas Research Institute of America LLC, Skokie, IL, USA

**Keywords:** animal model, bipolar disorder, depression, hippocampus, neural stem Cell, neurogenesis, schizophrenia

## Abstract

Several psychiatric and neurological diseases are associated with altered hippocampal neurogenesis, suggesting differing neural stem cell (NSC) function may play a critical role in these diseases. To investigate the role of resident NSCs in a murine model of psychiatric disease, we sought to isolate and characterize NSCs from alpha-calcium-/calmodulin-dependent protein kinase II heterozygous knockout (CaMK2α-hKO) mice, a model of schizophrenia/bipolar disorder. These mice display altered neurogenesis, impaired neuronal development and are part of a larger family possessing phenotypic and behavioral correlates of schizophrenia/bipolar disorder and a shared pathology referred to as the immature dentate gyrus (iDG). The extent to which NSCs contribute to iDG pathophysiology remains unclear. To address this, we established heterogeneous cultures of NSCs isolated from the hippocampal neuropoietic niche. When induced to differentiate, CaMK2α-hKO-derived NSCs recapitulate organotypic hippocampal neurogenesis, but generate larger numbers of immature neurons than wild-type (WT) littermates. Furthermore, mutant neurons fail to assume mature phenotypes (including morphology and MAP2/calbindin expression) at the same rate observed in WT counterparts. The increased production of immature neurons which fail to mature indicates that this reductionist model retains key animal- and iDG-specific maturational deficits observed in animal models and human patients. This is doubly significant, as these stem cells lack several developmental inputs present *in vivo*. Interestingly, NSCs were isolated from animals prior to the emergence of overt iDG pathophysiology, suggesting mutant NSCs may possess lasting intrinsic alterations and that altered NSC function may contribute to iDG pathophysiology in adult animals.

## Introduction

Altered neurogenesis (including altered proliferation, progeny development and morphology of stem cell-derived mature cell types) is reported in several neurological and psychiatric disorders, including epilepsy,^1^ autism,^2^ schizophrenia, Alzheimer's disease,^3^ Huntington's disease^3^ and depression,^4, 5^ among others. A common theme in these diseases are varying forms of cognitive impairment, many of which are linked to hippocampal function. As such, identification of common factors involved in neurogenesis and hippocampal function remains of high interest.^3^ In this study, we test the hypothesis that neural stem cells (NSCs) derived from an altered-neurogenesis mouse model of schizophrenia/bipolar disorder retain and recapitulate key disease features observed *in vivo*.

The immature dentate gyrus (iDG) is an emerging phenotypic feature conserved among several mouse models of schizophrenia/bipolar disorder (reviewed in Ref. 6), as iDG animals recapitulate many features of these diseases. Animals exhibit behavioral alterations frequently associated with schizophrenia/bipolar disorder, including hyperactivity, decreased spatial working memory, home-cage activity, cycling and nest-building deficits^7^ In the brain, these mice exhibit a conserved pathophysiological alteration termed the immature dentate gyrus. Interestingly, this feature is not present at birth, but appears to emerge in a period corresponding to late adolescence in at least two iDG mouse models (Ref. 8 and present study). Common alterations between iDG mice and human schizophrenia patients include hypoGABAergic phenotype, chronic alteration of inflammatory pathways and altered expression of calcium-binding proteins. More recently, key features of the iDG have been detected in postmortem samples from human schizophrenia and bipolar patients.^9^

CaMK2α-hKO mice are one of the oldest transgenic mouse models.^10^ Mutant mice display altered long-term potentiation^10^ and impaired spatial learning,^11^ a task associated with hippocampal function. CaMK2α-hKO mice possess constitutively higher levels of neurogenesis and display elevated levels of calretinin, PSA-NCAM and doublecortin and reduced levels of mature neuronal markers, most notably calbindin.^7^ Many iDG models also display hypoGABAergic function and altered numbers of interneurons, key features in schizophrenia/bipolar disorder. These features form the core of iDG pathophysiology (reviewed in Ref. 6). In recent years, iDG has been identified in an increasing number of chemical and genetic mouse models,^7, 8, 12, 13, 14, 15^ and features of the disease have been found in human schizophrenia/bipolar disorder patients.^9^

Altered neurogenesis in iDG animals is of particular interest as a potential contributor toward the hippocampal dysfunction and associated cognitive deficits. Several hypotheses address the contribution of altered neurogenesis to hippocampal dysfunction in schizophrenia/bipolar disorder. For example, one study suggests that constitutively elevated neurogenesis may be a contributor to the elevated oxidative stress observed in schizophrenia/bipolar patients,^16^ while other groups suggest that a lack of mature neurons (as observed in iDG mice) may contribute to a feedback loop where mature interneurons regulate NSC activity.^17, 18^ Regardless, characterizing altered neurogenesis in a reductionist cell culture model may provide new means to efficiently study key cells and processes in altered-neurogenesis mouse models of psychiatric disease.

To compare intrinsic differences in neurogenesis, we isolated NSCs from CaMK2α-hKO mice and their wild-type (WT) littermates. NSCs from both groups generated equivalent numbers of multipotent neurospheres, suggesting equivalent self-renewal and multipotency. However, when we injected BrdU to label dividing cells in WT and CaMK2α-hKO mice we found increased hippocampal and subventricular (SVZ) proliferation, suggesting that neurogenesis is constitutively elevated in mutant mice.

Next, in an attempt to identify alterations during neurogenesis in mutant NSCs, we established two-dimensional adherent cultures of hipppocampal NSCs derived from early postnatal CaMK2α-hKO mice and their WT littermates. NSCs cultured in this way can be induced to synchronously and terminally differentiate in a manner that recapitulates *in vivo* neurogenesis.^19^ We examined the proliferation patterns of WT and mutant mice before and after induction of differentiation. Undifferentiated NSCs from mutant mice possessed somewhat higher rates of proliferation and also expressed constitutively higher levels of proliferation in a 72 h period following induction of differentiation. This increased proliferation appeared to be focused on the generation of new neurons, as mutant NSCs transiently generate a larger number of neurons than their WT counterparts. However, these neurons fail to mature, as they progressively hypoexpress mature markers, including MAP2 and calbindin. Mutant neurons also display a stunted morphology and display elevated calretinin and reduced calbindin compared with developmentally-matched WT neurons. Collectively, these features match the hallmark features of iDG *in vivo*, indicating that this altered pathophysiology is retained in NSCs outside of the neuropoietic niche and that ongoing neurogenesis likely contributes to iDG features identified *in vivo*.

To examine the role of non-neuropoietic cells in neurogenesis, we examined mutant mice for iDG at the stage during which NSCs are harvested. Interestingly, early neonatal mice (the age at which NSCs are collected) do not appear to express iDG features. This is particularly relevant, as NSCs from mutant mice recapitulate key features of iDG-specific neurogenesis, even when removed from the larger cellular macroenvironment. Retention of altered neurogenesis in a reductionist model also allows for the possibility that altered neurogenesis may be intrinsically retained in mutant mouse NSCs, although additional experiments are needed to confirm this. Collectively, these findings demonstrate that stem cells derived from an animal model of psychiatric disease retain key features observed *in vivo*.

## Materials and methods

### Animal handling

Young (3–6-day-old) male and female C57/Bl6 and CaMK2α-hKO mice were used for NSC isolation (older animals were suitable for NSC isolation, albeit with reduced yields). 1- and 12-week-old male animals were used for *in vivo* studies. CaMK2α-hKO mice were generated and described previously.^10^ Prior to killing, unweaned pups were group housed under standard housing conditions. Animals were labeled, genotyped and immediately processed as groups (*n*=6 animals per group) of WT and mutant mice. For NSC isolation, killed animals were rapidly decapitated and brains were processed as described.^19, 20^ All protocols involving animals were developed and conducted in accordance with institutional IACUC protocols. Genotyping was performed using a 3-primer reaction with the following primers: Camk2a: forward, 5′-GTCAACTCAAACTCCACTGTCTCCAGGAAGGC-3′, reverse, 5′-CGTGGACTGCCTGAAGAAGTTCAATGCC-3′ Neo: reverse, 5′-GCGTTGGCTACCCGTGATATTGCTGAAGAG-3′.

### NSC Isolation/propagation/differentiation and neurosphere assay

For isolating, expanding and differentiating NSC/progenitor cells from the rodent hippocampus, we employed a protocol modified from Scheffler *et al.*^19^ and Babu *et al.*^20^ Cortical forebrain astrocytes were isolated from distal somatosensory cortex and processed identically. Briefly, animals (*n*=6 per group) were rapidly decapitated. Tissues were placed in 3X antibiotics (Invitrogen, Grand Island, NY, USA). Tissue was manually dissociated with a scalpel into 1 mm^3^ pieces, then placed into antiadhesive 100-mm culture dishes (Corning; Corning, NY, USA). Tissue was dissociated into a single-cell suspension using a commercially available papain-based neural dissociation kit (Miltenyi; Cologne, Germany). Samples were resuspended in PBS (Invitrogen) and filtered through a 40-μm cell strainer to obtain a final solution.

For establishment and propagation of adherent cultures, hippocampal NSCs were counted and seeded at identical densities on plastic dishes coated with poly-D-ornithine and laminin (BD Biosciences; Bedford, MA, USA). Growth medium consisted of Neurobasal medium supplemented with B27 supplements (Invitrogen), 2 mM Glutamax (Invitrogen), 1X penicillin/streptomycin, epidermal growth factor (20 ng ml^-1^; R&D Systems; Minneapolis, MN, USA), basic fibroblast growth factor (20 ng ml^-1^; R&D Systems) and bovine pituitary extract (0.5% v/v; Invitrogen). Growth factors were supplemented every other day in decreasing concentration (20 μl, 15 μl and 10 μl maintenance). Cells were passaged with 0.05% trypsin every 3–4 days. For neurogenesis studies, NSCs were plated onto glass coverslips coated with poly-D-ornithine and laminin (NeuVitro, El Monte, CA, USA) at a density of 2.5 × 10^5^cells cm^-2^ overnight in defined growth medium. To induce differentiation, serum, EGF and bFGF were removed from the culture media. During differentiation, a 50% media change was performed every third day.

Following each defined period of differentiation, cells were fixed for analysis with 4% formaldehyde for 15 minutes, after which they were transferred to PBS and stored at 4°C.

For clonal NSC analyses, primary neurospheres were isolated from dissociated ventricular neuraxis as described for adherent cultures. Following being passed through a cell strainer, live cells were counted on a hemacytometer using trypan dye exclusion. Cells were plated in antiadhesive 2.5-cm dishes (Corning; Corning, NY, USA) at a density of 1 × 10^3^ cells cm^-2^ in defined growth medium containing 1% methylcellulose, EGF and bFGF (both 20 ng ml^-1^). Six technical replicates were carried out for each experiment. Media was exchanged every 3 days. Primary neurospheres were dissociated into secondary spheres using commercially available reagents (Neurocult; Stem Cell Technologies; Vancouver, Canada). Hippocampal NSC/NPCs are not believed to generate neurospheres^21^ and were not analyzed for sphere formation. Neurosphere generation was expressed as average normalized to WT±s.e.m. Neurosphere diameter was measured using Keyence proprietary software.

### Immunohistochemistry/immunocytochemistry

For immunohistochemistry, animals were anesthetized with ketamine/xylazine and perfused with 10 ml of ice-cold 4% paraformaldehyde. Brains were removed and placed in 4% paraformaldehyde at 4°C overnight, then transferred to a 30% sucrose solution at 4°C for 3 days. Serial 30 μm coronal sections were cut on a Leica CM 3050 S freezing microtome (Leica Microsystems; Wetzlar, Germany) and stored at 4°C in cryoprotectant (0.1 M phosphate buffer containing: sucrose (30% w/v), ethylene glycol (30% v/v), polyvinyl-pyrrolidone (PVP-40; 1% w/v). For analysis, sections were removed from cryoprotectant, washed in phosphate buffered saline (PBS, pH 7.4). For BrdU detection, samples were pretreated with 1% SDS when necessary for the antibody used. Samples were blocked for 1.5 h at room temperature in PBS containing 0.03% Triton X-100, 3% normal donkey serum and 1% bovine serum albumin. Primary antibodies included: calretinin (rabbit polyclonal, 1:1000; Swant; Bellinoza, Switzerland), calbindin (rabbit polyclonal, 1:1000; Swant), pCREB (Ser133) (rabbit polyclonal, 1:500; Cell Signaling Technologies; Billerica, MA, USA), BrdU (rat monoclonal, 1:500; Abcam; Cambridge, MA,USA), doublecortin (rabbit polyclonal, 1:600; Cell Signaling Technologies), MAP2 (chicken polyclonal, 1:700; Aves Labs, Tigard, OR, USA), NeuN (mouse monoclonal, 1:500; Millipore; Billerica, MA, USA), PSA-NCAM (mouse monoclonal, 1:400; Millipore) and Tuj1 (β-III-tubulin; chicken polyclonal, 1:1,000; Aves Labs). Primary antibodies were applied overnight on an orbital shaker at 4°C. Samples were washed three times in PBS, then incubated with secondary antibodies on an orbital shaker for 75 min at room temperature. Secondary antibodies included goat anti-rat, Cy2/3-conjugated donkey anti-mouse, Cy2/3-conjugated donkey anti-rabbit (1:600; Jackson Immunoresearch; West Grove PA, USA). Primary and secondary antibodies were diluted in blocking solution. Samples were washed three times in PBS and mounted on glass slides. For BrdU imaging, samples were pretreated with sodium chloride/sodium citrate (SSC)-formamide (1:1, 37C, 2 h), washed three times for 10 min in SSC, incubated in 2 N HCl (37°C, 30 min) and washed with 0.1 M borate buffer (25 °C, 10 min). Adherent NSC/NPCs were handled as described, seeded onto glass coverslips coated with poly-D-ornithine and laminin, fixed with 4% paraformaldehyde and labeled as described for tissue sections. Labeled samples were mounted in mounting media containing DAPI (Vector Labs, Burlingame, CA, USA) and visualized on a BZ-9000 microscope (Keyence; Chicago, IL, USA). BrdU-positive cells were manually counted and expressed as cells/section. Data was analyzed using Graphpad software (La Jolla, CA, USA).

For quantitative immunocytochemistry, expression of MAP2, NeuN, pCREB, PSA-NCAM and Tuj1 were measured in undifferentiated NSCs and in NSCs induced to differentiate for 3, 7 and 14 days. Nine fields were counted per condition in an equally spaced ‘X' pattern. Three technical replicates were performed for each marker and condition. All samples were harvested and processed for ICC in parallel. Expression of each marker was measured using matched intensity exposures and quantitated using ImageJ (National Institute of Health). Lineage marker intensity was quantified using total signal/field, normalized to total cell number using the following equation: Normalized Field Intensity=(Field Intensity/DAPI-positive Cell Number). DAPI-positive cell counts were made on a Keyence BZ-9000 using proprietary quantitative software. For BrdU quantification in differentiating NSCs, 10 μM BrdU was added to matched cultures in non-overlapping 24 h periods. BrdU-positive cells were counted manually using the previously-described approach and expressed as a fraction of total DAPI-positive cell number. Golgi staining was performed on freshly isolated whole adult brains using commercial reagents (FD Neurotechnologies; Columbia, MD, USA) and mounted in 350 μm sections. TUNEL staining was performed on fixed tissues using commercially available reagents (Life Technologies, Carlsbad, CA, USA).

## Results

*In vivo*, CaMK2α-hKO mice exhibit elevated neurogenesis (Ref. 7 and Supplementary Figure S1). To determine whether this is the result of enhanced NSC activity or increased numbers of stem progenitor populations, we examined the prevalence of NSCs in the brains of CaMK2α-hKO mice and WT littermates using the neurosphere challenge assay. Ventricular neuraxis (including hippocampus and SVZ) was dissociated to single-cell suspensions and subjected to a neurosphere challenge assay as a means of quantifying self-renewing, multipotent NSCs.^22^ Dissociated WT and mutant cells formed primary and secondary neurospheres at roughly the same frequency (Supplementary Figure S1a) and size (Supplementary Figure S1b), indicating comparable NSC numbers and equivalent self-renewal. As only hippocampal neurogenesis has been described as being elevated in CaMK2α-hKO mice and SVZ NSCs are generally acknowledged to be the only neuropoietic population capable of neurosphere formation,^23^ we examined the lateral subependymal cell layer for proliferation following short-term (24 h) 5-bromodeoxyuridine (BrdU) labeling. We observed a significant increase in BrdU-labeled SVZ cells (Supplementary Figures S1c-e) in CaMK2α-hKO mice, suggesting that (previously undescribed) SVZ proliferation is also elevated in this model. Hippocampal proliferation was similarly increased in these mice (Supplementary Figure S1f), suggesting hyperneurogenesis occurs in all postnatal neuropoietic niches. Stem/progenitor cells from WT and CaMK2α-hKO mice generated neurons, astrocytes and oligodendrocytes from both neurospheres and adherent cell cultures (Figure 2 and data not shown).

**Figure 1:**
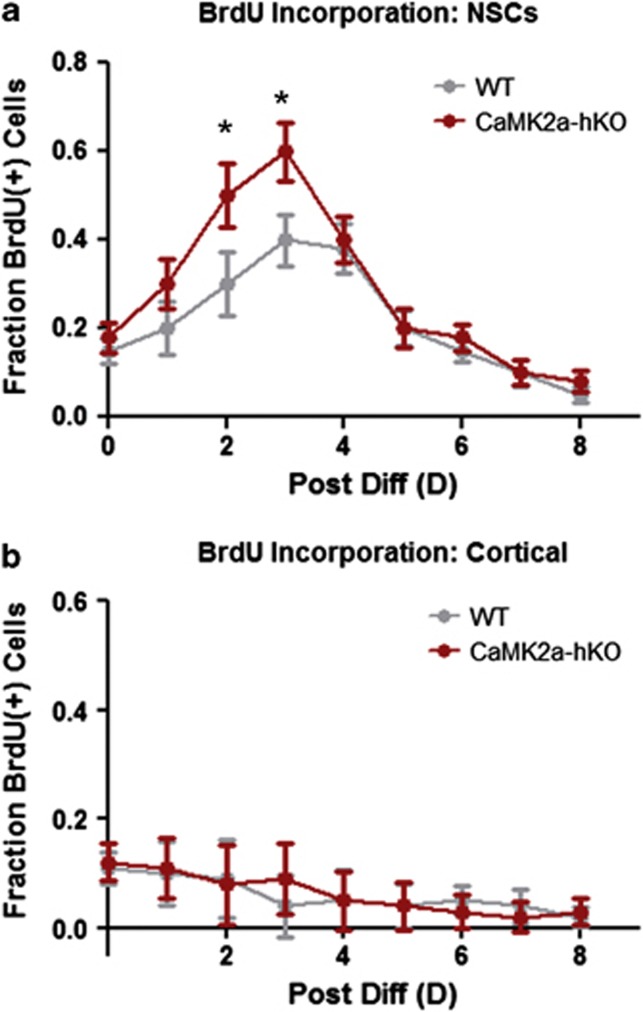
Proliferation of NSCs throughout differentiation. (**a**) CaMK2α-hKO-derived NSCs exhibit increased proliferation 1–3 days after initiation of differentiation. (**b**) Identically cultured cortical astrocytes fail to show differences in proliferation or alteration of proliferation rates in response to mitogenic withdrawal. *P<0.05, unpaired Student's *t*-test.

Based on these results, we next compared stem/progenitor function in WT and mutant mice by isolating undifferentiated stem/progenitor cells and comparing their proliferative kinetics during differentiation. Undifferentiated NSCs can be induced to terminally differentiate following removal of essential mitogenic factors,^19^ a process that closely follows organotypic differentiation observed *in vivo*^19, 24^ and allows for direct comparison of WT and mutant stem/progenitor populations. We first examined NSC and progenitor proliferation during the generation of progeny by pulse-labeling differentiating NSCs using BrdU in consecutive, non-overlapping 24 h periods. As previously reported,^19, 24^ induction of terminal differentiation *in vitro* elicits a transient increase in the rate of cellular proliferation (measured by BrdU-positive cells) 1–4 days following differentiation. This increase likely represents the presence of highly active intermediate progenitors, which give way to increasingly quiescent neuroblasts and postnatal neurons.^25, 26^ We appreciated this characteristic increase in proliferation in WT NSCs (Figure 1a). However, mutant NSCs exhibit larger (and possibly more immediate) increases in proliferation than WT counterparts (Figure 1a). To determine if this was related to perpetually elevated activity of multipotent NSCs, we compared basal proliferation rates of undifferentiated WT and mutant NSCs for five passages. Mutant NSCs exhibited a higher (insignificant) increases in proliferation (0.416±0.05 vs 0.356±0.04 doublings per day, *P*=0.03/0.06, one/two-tailed *t*-test), suggesting their basal activity is somewhat higher. This proliferative increase appears to be restricted to neuropoieic cells, as non-neurogenic cortical astrocytes subjected to differentiate did not display increased proliferation over WT cortical astrocytes (Figure 1b) or alter levels of proliferation in response to induction of differentiation (Figure 1b).

**Figure 2:**
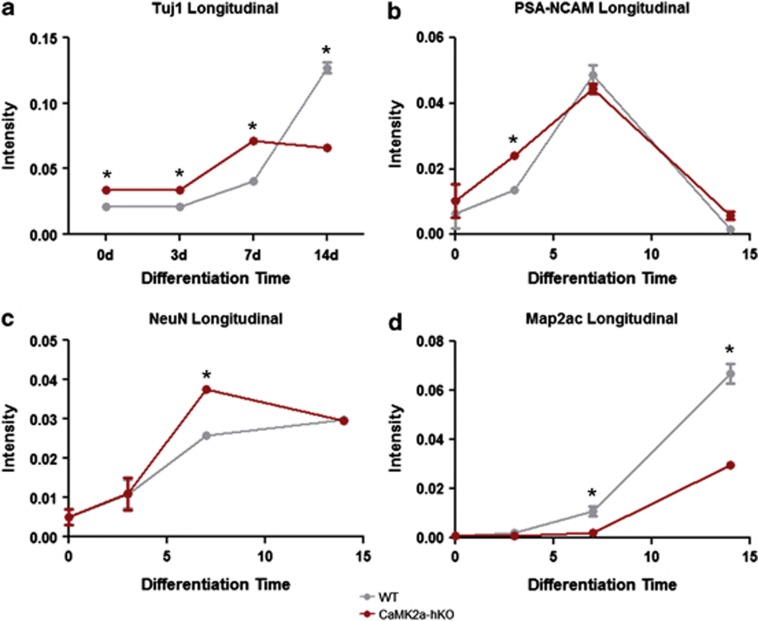
Expression of early-, late- and pan-neurogenic markers throughout differentiation of WT- and CaMK2α-hKO-derived NSCs. (**a**) Mutant NSCs initially express elevated levels of the pan-neurogenic marker Tuj1, which is maintained 3 and 7 days after differentiation. This is matched by increased expression of the early neuron marker PSA-NCAM 3 days after initiation of differentiation (**b**). (**c**) Mutant-derived NSCs generate significantly higher numbers of NeuN-positive mature neurons 7 days after differentiation (which is not sustained when examined at 14 days). (**d**) 7 day after differentiation, mutant NSCs begin to hypoexpress the mature neuronal marker MAP2, which is further decreased at 14 days. Expression is measured as by total immunofluorescent signal divided by total cell number. *P<0.05, unpaired Student's *t*-test.

Next, we compared the endogenous potential of mutant and WT NSCs to generate neurons by measuring neuronal production following differentiation. Using quantitative immunocytochemistry, we measured the expression of the pan-neuronal marker Tuj1, immature neuron marker PSA-NCAM and the mature neuronal markers NeuN and MAP2 prior to differentiation and at 3, 7 and 14 days after differentiation (developmental periods corresponding to immature neurons/neuroblasts, early postmitotic neurons and mature neurons). WT and mutant NSCs initially express low levels of neuron-specific proteins (Figures 2a and d), agreeing with previous findings suggesting the preponderance of undifferentiated cell cultures are comprised of glial/gliotypic NSCs and other non-neuronal cells from the hippocampal niche.^16, 19, 24, 27^ When induced to differentiate, mutant NSCs generated larger numbers of neurons more rapidly, as evidenced by increased expression of Tuj1 (Figure 2a) and PSA-NCAM (Figure 2b) 3 days after initiation of differentiation. Mutant NSCs express markedly higher Tuj1 expression in undifferentiated cells (Figure 2a; Tuj1 expression has previously been appreciated in gliotypic/undifferentiated NSCs, as described in Refs. 24,28), reaching maximum expression 7 days following differentiation. In contrast, WT NSCs progressively increase Tuj1 expression, but temporally trail mutants in the expression of this pan-neuronal marker (Figure 2a). PSA-NCAM, which labels early neurons, is transiently expressed in both WT and mutant populations (Figure 2b), peaking 7 days after differentiation. PSA-NCAM expression is significantly higher in mutant animals 3 days after differentiation (Figure 2b), indicating neuronal generation occurs more rapidly in mutant progenitor populations. Together, these markers suggest that the immediate generation of new neurons in the first seven days following terminal differentiation occurs more definitively and more rapidly in mutant-derived NSCs.

We next measured the expression of the mature neuronal markers NeuN and MAP2. WT-derived NSCs generate steadily increasing numbers of NeuN-positive neurons at 3, 7 and 14 days (Figure 2c). Mutant NSC production of NeuN-positive neurons peaks at 7 days, then decreases (Figure 2c). It is unclear as to whether this is a global loss of NeuN or a loss of NeuN-positive neurons, as total NeuN expression in mutant mice decreases from 7 to 14 days, but total intensity remains comparable to WT 2 weeks after differentiation. MAP2 expression increases in WT and mutant-derived neurons as differentiation progresses. However, when compared with WT-derived counterparts, mutant-derived neurons express relatively lower levels of MAP2 beginning at 7 days and continuing at 14 days (Figure 2d). To assess cell death in WT and mutant-derived NSCs, we measured TUNEL staining in undifferentiated and differentiating NSCs. Undifferentiated NSCs did not express significantly different fractions of TUNEL-positive cells (Supplementary Figure S2). Mutant-derived NSCs did display increased TUNEL staining at 3 and 7 days after differentiation (Supplementary Figure S2), but these differences were not significant when normalized to the numbers of neurons (Tuj1-positive cells) produced (Supplementary Figure S2).

**Figure 3:**
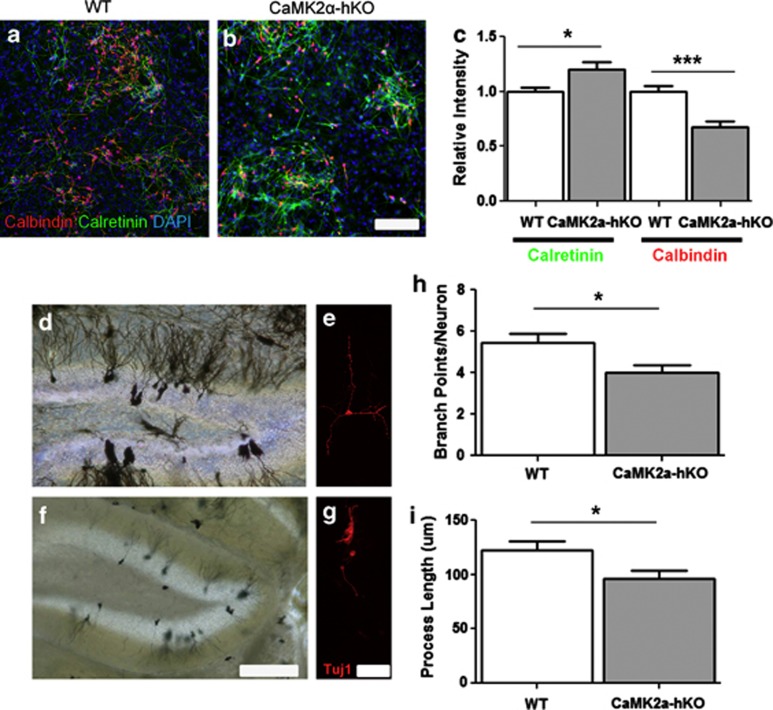
Mutant-derived neurons display iDG-like features. (**a**) Twenty-one-day-old CaMK2α-hKO NSC-derived neurons display decreased calbindin (**b**) and increased calretinin (**c**) when compared with WT counterparts. *In vivo* Golgi staining reveals CaMK2α-hKO mice display stunted neuronal morphologies (**f**) compared with WT (**d**) counterparts. NSC-derived neurons from these mutants also possess a stunted, less processive morphology (labeled with Tuj1; **e**, **g**), and display fewer dendritic branches (**h**) and shorter processes (**i**). Scale bar=100 μm (**a**, **b**), 150 μm (**d**, **f**), 50 μm (**e**, **g**). Calbindin/calretinin expression is measured as total immunofluorescent signal divided by total cell number and normalized to WT expression. *P<0.05, ***P<0.001, unpaired *t*-test.

A hallmark phenotype of CaMK2α-hKO and other iDG mice is the unbalanced expression of key calcium-binding proteins in neurons throughout the hippocampus.^7^ In particular, the expression of the immature neuronal marker calretinin is increased in the subgranular zone and dentate gyrus and the mature neuronal marker calbindin is hypoexpressed throughout the granule cell layer.^7^ As it is unclear as to whether ongoing postnatal neurogenesis contributes to these aberrant populations, we measured the expression of calretinin and calbindin in 21-day-old neurons generated by WT and mutant NSCs. In normal neurogenesis, calretinin expression precedes calbindin expression. WT-derived NSCs generated calretinin and calbindin-positive neurons, which frequently co-express both markers, presumably recapitulating the overlapping period of expression during development (Figure 3a). We compared the numbers of calretinin and calbindin cells generated by WT and mutant mice at this time point using quantitative immunocytochemistry. As observed *in vivo*, CaMK2α-hKO-derived NSCs generated higher numbers of calretinin-positive cells and lower numbers of calbindin-positive cells as measured by total signal intensity (Figure 3c), suggesting a recapitulation of a neuron-specific iDG phenotype *in vitro*. The characteristic alteration in calretinin/calbindin are also retained when measured as a fraction of total cells or as in terms of signal intensity/positive cell number (data not shown), suggesting the measured expression changes are accomplished by changes in the total positive cell number and altered expression intensity of each marker.

Previous work has also indicated aberrant structure in granule cell neurons in human schizophrenia patients,^29^ a feature shared by CaMK2α-hKO mice (Ref. 9 and Figures 3d and f). As Golgi staining-based study cannot determine whether these neurons are generated postnatally, we examined the morphology of 21-day-old neurons generated from WT and CaMK2α-hKO NSCs. As seen *in vivo*, mutant-derived neurons possess a visibly stunted morphology with simpler, less branched processes (Figures 3e and g) when compared with WT neurons. We assessed the total number of dendritic branch points and the total process length on individual neuronal cells (*n*=72 and 64 WT and mutant, respectively, Figures 3h and i). Mutant neurons displayed significantly shorter processes with fewer branch points. From these experiments, we were able to determine that postnatally-generated neurons are a source of aberrant neurons observed in mutant mice.

**Figure 4:**
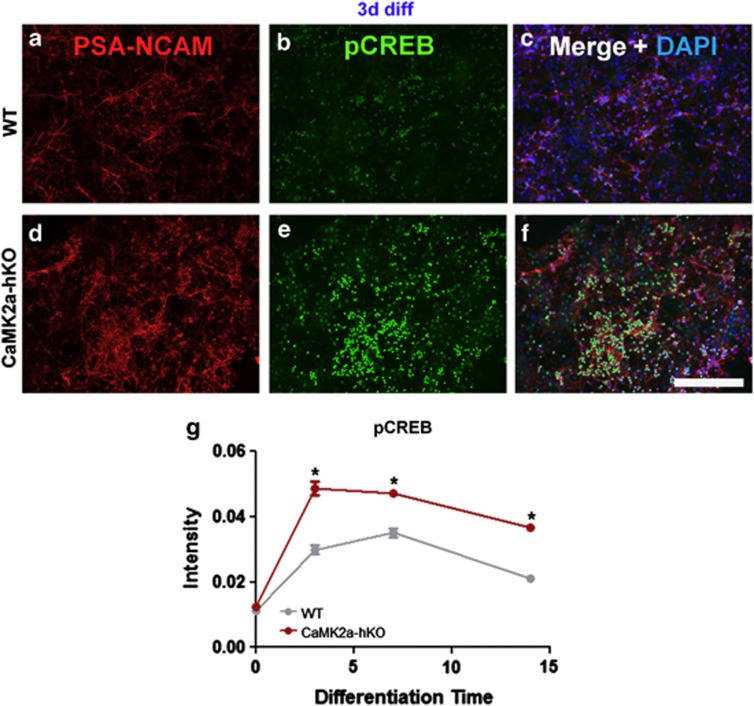
pCREB expression is hyperexpressed in CaMK2α-hKO-derived NSCs following differentiation. (**a**-**f**) Differentiating clusters of PSA-NCAM-labeled neurons display increased pCREB in exposure-matched images 3 days after differentiation. (**g**) pCREB is significantly upregulated in CaMK2α-hKO-derived progenitors, immature- and mature neurons following differentiation. Total pCREB signal normalized to cell number. *P<0.05, unpaired *t*-test. Scale bar=100 μm.

Neurogenesis regulation is regulated by a multifaceted and complex series of regulatory molecules. CaMK2α-hKO mice have shown alterations in many key genes, including *Arc*, *c-fos* and *pCREB.*^7^ In particular, pCREB is present in both the hippocampal and SVZ neuropoietic niches and is expressed transiently throughout neuronal development (reviewed in Ref. 30). Moreover, modulation of CREB signaling ameliorated key cellular and behavioral deficits in other iDG models.^8^ We investigated the expression of pCREB(Ser133-specific) in differentiating WT and mutant NSCs. Following differentiation, pCREB expression is transiently elevated, reaching a maximum 3 days after differentiation and decreasing as neuronal maturation continues (Figure 4g). This matches reports that CREB signaling is transiently increased during *in vivo* neurogenesis.^30^ Differentiating CaMK2α-hKO NSCs strongly hyperexpress pCREB in newly generated neurons (Figures 4a and f) and throughout all stages of neurogenesis measured (Figure 4g).

**Figure 5:**
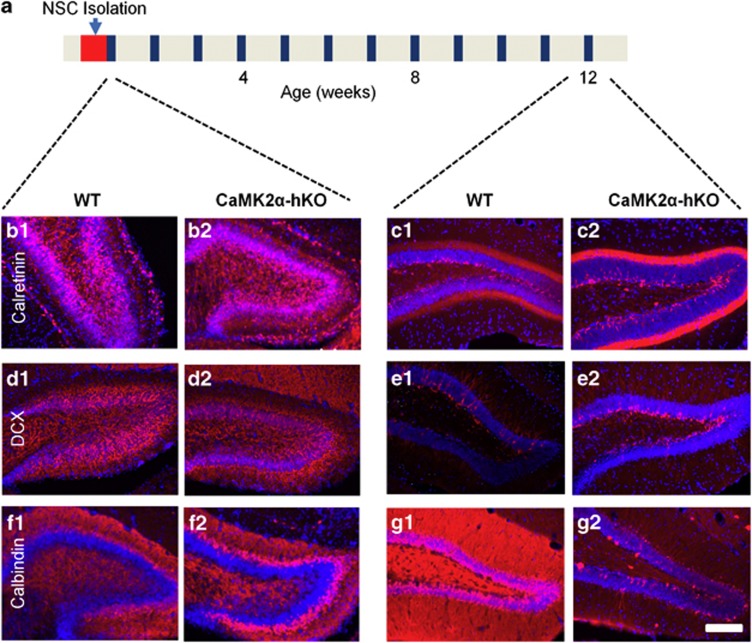
(**a**) Timeline of development indicates points of NSC isolation (postnatal day 3–6) and sacrifice points of WT and CaMK2α-hKO mice (1- and 12-weeks-old, respectively) for examination of classic iDG hallmarks. We examined calretinin (**b**, **c**), doublecortin (**d**, **e**) and calbindin (**f**, **g**). No difference in marker expression was detected in 1-week-old animals (**b1**/**d1**/**f1** v **b2**/**d2**/**f2**). Classic iDG hallmarks were observed in all three markers at 12 weeks of age, including increased doublecortin (**c1** v **c2**) and calretinin (**e1** v **e2**) and decreased calbindin (**g1** v **g2**). Scale bar=100 μm.

Finally, we sought to examine whether the observed alterations in CaMK2α-hKO neurogenesis are intrinsically programmed or are a product of cellular microenvironment (that is, existing circuit integration, endothelial cellsand so on). To do this, we sought to determine whether iDG hallmarks are present when we isolated NSCs. To accomplish this, we compared pathophysiological iDG hallmarks in 1- and 12-week-old mice (the ages at which we isolated NSCs and when iDG hallmarks are well established). 1-week-old CaMK2α-hKO mice were indistinguishable from WT mice for immature markers calretinin (Figures 5b and c) and doublecortin (Figures 5d and e), as well as the mature neuronal marker calbindin (Figures 5f and g). As these markers are dysregulated in 12-week-old animals, it appears that predisposing alterations CaMK2α-hKO NSCs occur before iDG emergence and may contribute to the initial development and maintenance of iDG pathophysiology.

## Discussion

While the exact role of neurogenesis in various psychiatric and neurological diseases remains unclear and remains contested,^31^ increasing numbers of studies suggest that neurogenesis may be altered in a number of CNS diseases and disorders. Furthermore, studies point to the ability of several common CNS therapeutics (most notably SSRIs) to modulate neurogenesis.^32^ As such, the function of NSCs in disease models is of particular interest. We sought to examine NSC function in an animal model of schizophrenia/bipolar disorder that displays altered neurogenesis.

We sought to identify endogenous capabilities and behaviors of NSCs isolated from CaMK2α-hKO mice, a member of a family of mutants possessing dentate gyrus immaturity (including increased neurogenesis) as well as phenotypic alterations (altered GABAergic and inflammation status) and conserved behavioral correlates (social interaction deficits and cognitive impairment) of schizophrenia/bipolar disorder. Using an established *in vitro* model of NSC culture, we were able to compare the characteristics of mutant-derived NSCs to WT counterparts, including the recapitulation of neurogenesis.

Mutant animals displayed equivalent numbers of multipotent, self-renewing NSCs, but displayed elevated proliferation *in vivo* and *in vitro*. This may represent a compensatory effect for a lack of developed neurons (a common theme among iDG mouse models), intrinsic NSC alteration or another form of altered NSC regulation from non-neuropoietic cells. Moreover, the increased activity (particularly observed after neurogenesis induction) of mutant NSCs may have functional consequences relevant to schizophrenia pathology. For example, neurogenesis has been linked to the generation of oxidative stress, particularly during highly mitotic phases.^16^ Other studies indicate that localized oxidative stress in perineuronal networks may lead to impaired hippocampal function.^33, 34, 35^ Human schizophrenia patients possess smaller, possibly atrophied, hippocampi^36^ with altered cell morphology,^37^ possible signs of oxidative atrophy. Although we did not measure indicators of oxidative stress in this study, the previously established relationship between neurogenesis and oxidative stress suggests oxidative species are generated in a proliferation-dependent manner, strongly suggesting excessive redox stress may occur in mutant NSCs. Interestingly, we also appreciated elevated SVZ neurogenesis in CaMK2α-hKO mice, suggesting the possibility that comparable deficits in olfaction may exist in this model, possibly through the same mechanisms. While human schizophrenia/bipolar disorder patients often display deficits in olfaction,^38, 39^ such a conclusion remains contested in light of conflicting studies over the extent of human SVZ neurogenesis.^40, 41, 42^

*In vivo*, CaMK2α-hKO and other iDG mice generate large numbers of immature neurons that do not mature into granule cell neuron phenotypes. *In vitro* study of mutant-derived NSC differentiation accurately recapitulated many of the factors observed *in vivo*: mutant NSCs produce larger numbers of early neurons, doing so more rapidly than WT counterparts. However, as differentiation progressed, these neurons began to express lowered levels of key maturational markers (including MAP2 and calbindin). Maturational markers that were not observed to be different *in vivo* were not significantly affected. Mutant-derived neurons developed stunted morphologies and expressed higher levels of calretinin, similar to *in vivo* observations. Total cell death was elevated following differentiation in mutant NSCs. However, these differences were not significant when normalized to the numbers of neurons produced, suggesting an equivalent rate of cell death in mutant-derived neurons. From these findings, we conclude that a failure to properly mature exists in CaMK2α-hKO-derived early neurons, likely contributing to the altered neuronal morphologies observed *in vivo*. Furthermore, we were able to detect these cells using a reductionist cell culture model that lacked many of the developmental cues of the hippocampal neuropoietic niche. That these alterations persist in a simplified culture system suggests intrinsic regulation of NSCs, rather than a dominating input from non-neuropoietic cells (for example, synapsing neurons). This is particularly interesting as CaMK2α is primarily expressed in neurons, yet alterations in proliferation rate are seen in mutant-derived NSCs and early neurons, which express CaMK2α at very low levels.

To further investigate the environment-versus-endogenous regulatory effect on NSCs, we sought to determine whether mutant NSCs isolated prior to iDG emergence retained the same properties for altered neurogenesis. iDG was not present in early postnatal mice; however, NSCs isolated from these mice retained characteristic iDG-like alterations to proliferation and maturation. This indicates that (a) iDG is not present at birth (and is presumably developed) and (b) mutant NSCs appear to retain altered properties from birth forward. While further experiments (notably the co-culture/co-transplantation of WT and mutant NSCs) are needed to examine the balance of internal and external inputs on mutant NSC function, our findings thus far suggest that mutant NSCs durably retain key traits both *in vivo* and outside their cellular microenvironment.

In summary, CaMK2α-hKO-generated neurons retain elevated neurogenesis and overexpress immature neuronal makers and then hypoexpress mature markers and assume simplistic, stunted morphologies. Postnatal neurogenesis likely contributes to the observed neuronal dysfunction in mutant mice, with mutant NSCs possessing altered capacities prior to the development of iDG hallmarks *in vivo*. As demonstrated with the study of iPS-derived neurons from psychiatric disease patients,^43^ the use reductionist culture systems is a powerful tool, both as a means of studying altered neurogenesis/neuronal development or as a screen for potential therapeutics. Similarly, recent progress in directly measuring human hippocampal neurogenesis^44^ should reveal the significance in altered rates of neurogenesis and neuronal development in various CNS disorders.

## Notes

All authors are employees of Astellas Pharma Inc., which provided support for these studies.
