# Supplementary figures and images for: Derivation of neural stem cells from an animal model of psychiatric disease
Source: Transl Psychiatry. 2013 Nov 5;3(11):e323–. doi: 10.1038/tp.2013.96 (PMC3849963; doi:10.1038/tp.2013.96)

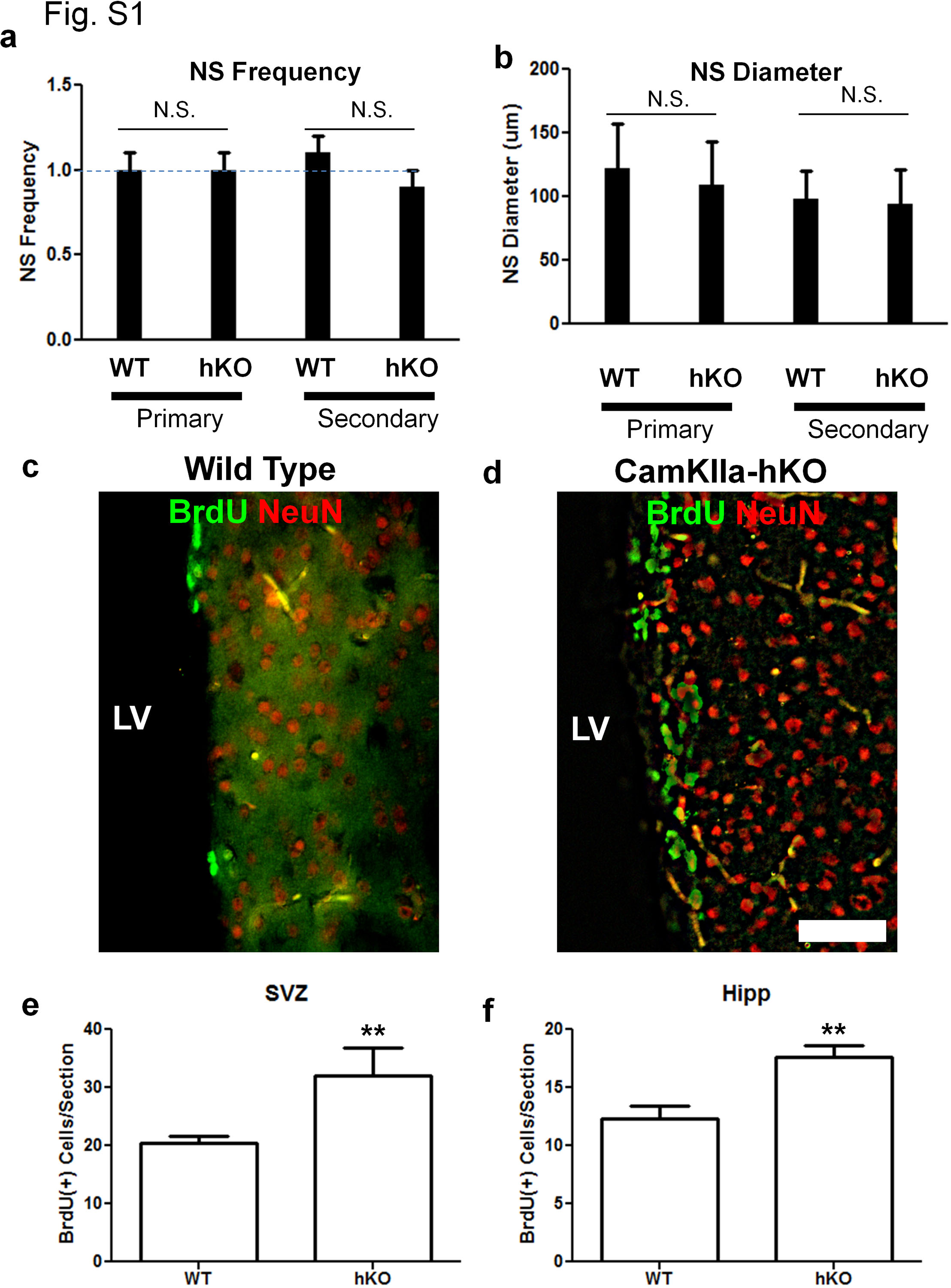

Supplement: Supplementary Figure S1 [file tp201396x1.tif]

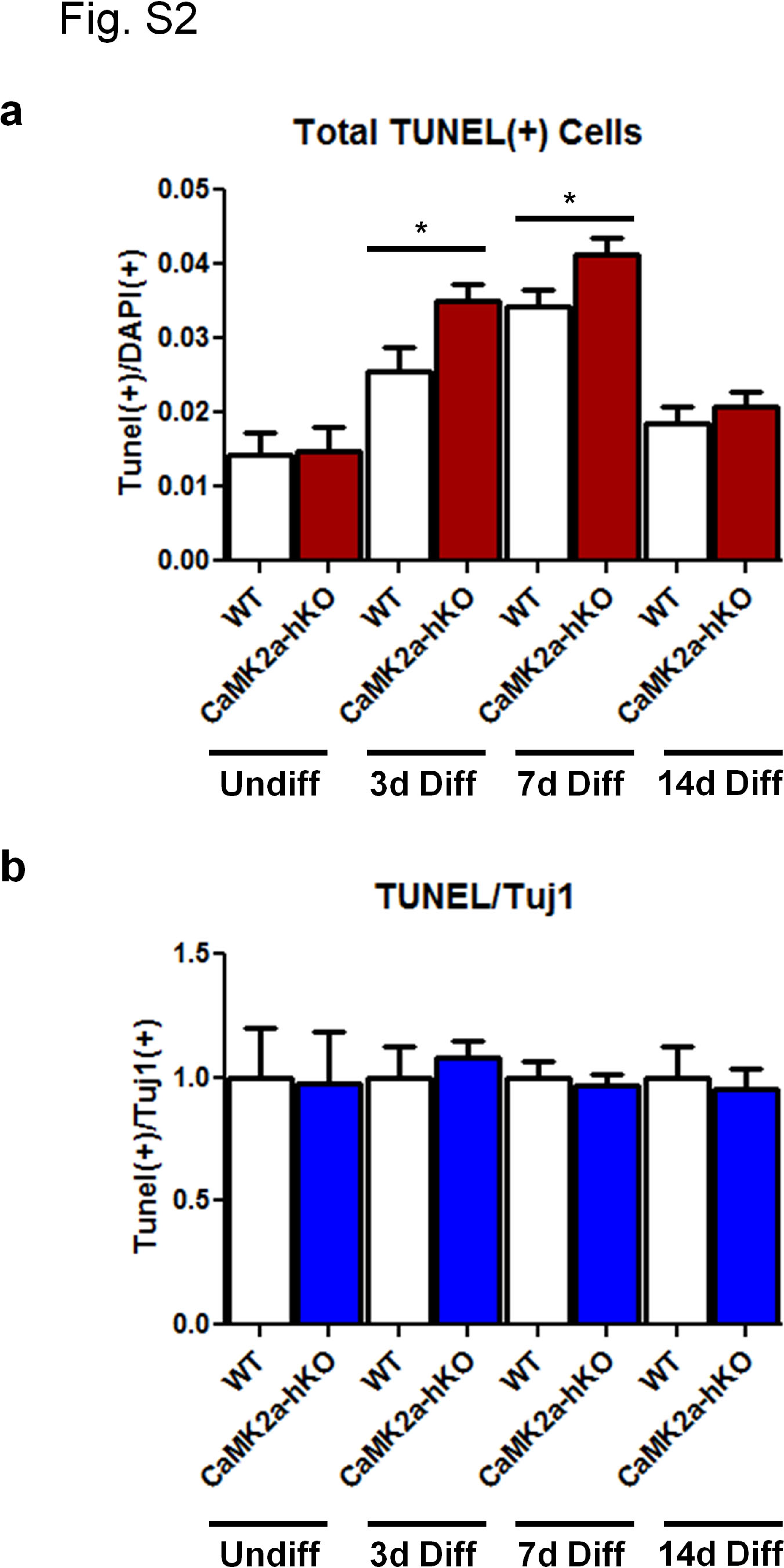

Supplement: Supplementary Figure S2 [file tp201396x2.tif]
