# Supplementary material for: Derivation of neural stem cells from an animal model of psychiatric disease
Source: Transl Psychiatry. 2013 Nov 5;3(11):e323–. doi: 10.1038/tp.2013.96 (PMC3849963; doi:10.1038/tp.2013.96)
Supplement: Supplementary Figures Legend [file tp201396x3.doc]

**Fig. S1.** WT and CaMK2α-hKO mice possess equivalent numbers of NSCs, but CaMK2α-hKO NSCs have increased proliferation. (a) Frequency of primary and secondary neurospheres (NS) generated following clonal seeding in anti-adherent conditions. (b) WT and mutant mice generate primary and secondary neurospheres of equivalent size. (c) BrdU-labeling of WT (c) and CaMK2α-hKO (d) supependymal cells following 24-hours after single BrdU injection. Proliferation is increased in CaMK2α-hKO mice in both the SVZ (e) and hippocampus (Hipp, f). Scale bar = 50 µm. p**<0.01, student’s t-test.

**Fig. S2.** TUNEL staining in differentiating WT and mutant-derived NSCs. (a) Total fraction of TUNEL-positive cells, normalized to total DAPI-positive cell number. (b) TUNEL cell number, normalized to Tuj1-positive (neuronal) cell number. Values in (b) are expressed as a fraction of WT for each condition. p*<0.05, student’s t-test.
